# Is it beneficial to use apertures in proton radiosurgery with a scanning beam? A dosimetric comparison in neurinoma and meningioma patients

**DOI:** 10.1002/acm2.13459

**Published:** 2021-11-09

**Authors:** Roberto Righetto, Francesco Fellin, Daniele Scartoni, Maurizio Amichetti, Marco Schwarz, Dante Amelio, Paolo Farace

**Affiliations:** ^1^ Proton Therapy Unit S. Chiara Hospital–Azienda Provinciale per I Servizi Sanitari (APSS) Trento Italy; ^2^ Trento Institute for Fundamental Physics and Applications (TIFPA) National Institute for Nuclear Physics, (INFN) Povo Italy

**Keywords:** apertures, pencil beam scanning, proton therapy, radiosurgery, robustness

## Abstract

**Purpose:**

To assess the dosimetric advantages of apertures in intracranial single fraction proton radiosurgery.

**Materials and methods:**

Six neuroma and 10 meningioma patients were investigated. For each patient, six plans were computed, with two spot spacing and three aperture settings (no apertures, 5 and 8 mm margin between aperture and clinical target volume [CTV]). All plans were optimized on the CTV with the same beam arrangement and the same single‐field robust optimization (2 mm setup errors, 3.5% range uncertainties). Robustness analysis was performed with 0.5 and 1.0 mm systematic setup errors and 3.5% range uncertainties. CTV coverage in the perturbed scenarios and healthy brain tissue sparing in the surrounding of the CTV were compared.

**Results:**

Meningiomas were larger and at a shallow depth than neuromas. In neuromas, spot spacing did not affect OAR doses or the robustness of CTV coverage and the apertures reduced brain dose without any significant impact on CTV robustness. In meningiomas, smaller spot spacing produced a reduction in brain V5Gy and improved robustness of CTV coverage; in addition, an 8 mm margin aperture reduced low and medium brain tissue doses without affecting robustness in the 0.5 mm perturbed scenario. A 5 mm margin aperture caused a reduction of plan robustness.

**Conclusion:**

The optimal use of apertures is a trade‐off between sparing of low and medium dose to the healthy brain and robustness of target coverage, also depending on size and depth of the lesion.

## 1 INTRODUCTION

Proton radiosurgery was pioneered in the 1960s at the Harvard Cyclotron Laboratory,^1^ and over the course of years has evolved with respect to both immobilization and imaging systems. Moreover, proton radiosurgery has also been affected by the changes in the beam delivery systems. Although fixed beam line can be adopted for radiosurgery treatments,^2^ the increased availability of gantries impacted proton‐based radiosurgery. Another major change in the proton delivery systems is the transition from passive scattering system^3^, ^4^ to pencil beam scanning (PBS).

The use of patient and field‐specific brass aperture blocks is usual in particle therapy delivered with the passive scattering technique. In PBS, dose optimization is based on fluence modulation and the proton beam is magnetically collimated. PBS radiosurgery performed with a small spot size can achieve a comparable or smaller risk of brain necrosis than photon radiosurgery^5^ and can reduce the integral dose to the healthy brain.^6^

The lateral penumbra may be improved in some situations with PBS using aperture blocks. The combination of PBS with such additional collimation has been investigated to assess the impact on plan quality^5^ and for superficial targets.^6^ The optimal method to select an aperture margin was also investigated.^7^

We are currently performing PBS radiosurgery for the treatment of selected acoustic neuromas and meningiomas without the use of aperture blocks. This study is aimed at assessing the potential advantages and disadvantages of apertures in PBS proton therapy for those two malignancies.

## 2 METHODS

### 2.1 Patient treatment planning

Patients with acoustic neuroma or meningioma who received proton PBS radiosurgery in our center were retrospectively investigated. The prescribed dose for neuromas and meningiomas was 12 Gy(RBE) and 15 Gy(RBE), respectively, delivered in a single fraction.

Patient contouring and treatment planning were performed in RayStation (Version 8.1.1, RaySearch Laboratories AB, Stockholm, Sweden). For each patient, the target volume and the adjacent organs‐at‐risk (OAR) were defined on the planning CT.

Plans were calculated considering a constant RBE of 1.1 and using a Monte Carlo dose‐engine to compute dose distribution (associated statistical uncertainty 1%) on a 1 mm^3^/voxel dose grid. For neuromas three beams were used, two oblique‐posterior and one non‐coplanar. For meningiomas, the location of the lesion did vary from patient to patient and so did the beams arrangement, with the number of used fields varying between two and three.

In our system (IBA Proteus One with dedicated nozzle), the spot size varies from 2.8 mm (one sigma in air at isocenter) for 226 MeV to 6.7 mm for 70 MeV. For shallow lesions, a 4 cm WET pre‐absorber was used to deliver the dose in the more superficial layers. The aperture is mounted on a movable snout upstream with respect to the pre‐absorber and both devices are positioned as close as possible to the patient surface.

Dose distributions were obtained by single field optimization, using min‐max robust optimization.^8^ All beams were required to individually ensure a uniform clinical target volume (CTV) dose, robust with respect to setup uncertainties of 2 mm and ±3.5% range uncertainties. In each plan, the dose was normalized so that 99% of CTV was covered by the prescribed dose (D99% = prescribed dose).

In all neuroma patients, the dose to 1% (D1) of brainstem, the V cranial nerve, and the healthy brain tissue was kept below 12 Gy(RBE). In one patient (patient #3), pre‐treatment hearing was not compromised so the average dose to cochlea was kept lower than 4 Gy(RBE).^9^ For the other patients, with compromised hearing, the D1 of the cochlea was kept below 12 Gy(RBE).

In the meningioma patients, the dose to the healthy brain tissue was minimized trying to keep the volume receiving more than 10 Gy(RBE) (V10Gy) less than 10 cc and the volume receiving more than 12 Gy(RBE) (V12Gy) less than 5 cc.

### 2.2 Variable spot spacing and margin: Investigated scenarios

In the treatment planning system (TPS), for each energy layer, the spot spacing is set to 1.06 times the spot size sigma for that energy^10^ and can be modified by the planner using a scaling factor. The spot spacing refers to the position of the spots at the beginning of the optimization. During the optimization, the TPS automatically remove all the spots with less than 0.02 MU, which is the minimum MU value deliverable by the treatment system. Aperture margin is defined as the distance in the beam's eye view between the CTV edge and the aperture.^5^

For each patient, the following six plans were optimized:

‐ S0.5 NA, spot spacing scaling factor equal to 0.5 and no aperture;
‐ S1.0 NA, spot spacing scaling factor equal 1.0 and no aperture;
‐ S0.5 M5, spot spacing scaling factor equal to 0.5 and aperture with a margin of 5 mm;
‐ S1.0 M5, spot spacing scaling factor equal to 1.0 and aperture with a margin of 5 mm;
‐ S0.5 M8, spot spacing scaling factor equal to 0.5 and aperture with a margin of 8 mm;
‐ S1.0 M8, spot spacing scaling factor equal to 1.0 and aperture with a margin of 8 mm.

### 2.3 Plan comparison and robustness analysis

Target coverage and the dose to the healthy brain tissue were evaluated for the six plans in the nominal plan and in two scenarios. In the first scenario, the nominal dose distribution was recomputed applying 0.5 mm shift of the isocenter in all directions (3D vector length = 0.9 mm). In the second scenario, a 1 mm shift was applied in all directions (3D vector length = 1.7 mm). In both scenarios, a range uncertainty of ±3.5% was simulated. The nominal and the two worst‐case scenarios were compared among the six plans according to the following dose parameters: CTV D99% for all target volumes, V5Gy, V10Gy, and V12Gy to normal brain tissue for neuromas, and V5Gy, V10Gy, V12Gy, and V15Gy for meningiomas. We used a *t*‐test to evaluate statistical significance. A *p*‐value lower than 0.05 was considered significant.

Finally, a gradient index (GI), defined as the ratio of the volumes covered by half of the prescribed dose and the volume covered by the prescribed dose,^11^ was quantified for each plan.

## 3 RESULTS

A total of 16 patients were identified, six of them affected by neuroma (neuroma patients # 1–6) and 10 by meningioma (meningioma patients # 1–10). The characteristics of the patients, the locations of the lesions, and the beam arrangements are reported in Table 1. The median CTV volume was 0.53 cc (range 0.03–0.83 cc) for neuromas and 1.58 cc (range 0.35–3.62 cc) for meningiomas (see patient details in Table 1).

**TABLE 1:** Volume [cc], location of the lesion (left or right for neurinomas and supero‐inferior, right‐left and anterior‐posterior planes for meningiomas), and beam arrangements

Neuroma			
Patient	Volume [cc]	Lesion site	Beam arrangement
1	0.70	Left	G200 C0
			G165 C0
			G90 C300
2	0.16	Left	G200 C0
			G165 C0
			G90 C310
3	0.79	Left	G205C0
			G155C0
			G210C90
4	0.83	Right	G170C0
			G200C0
			G270C50
5	0.03	Left	G200C0
			G165C0
			G90C310
6	0.64	Left	G2190C0
			G160C0
			G90C310
Menigioma			
1	0.84	SI: caudal	G80C0**
		RL: medial	G180C0**
		AP: medial	G285C0**
2	3.62	SI: medial	G65C0**
		RL: right	G165C0**
		AP: post	G205C0**
3	2.57	SI: medial	G270C0**
		RL: right	G180C0**
		AP: post	G270C90**
4	1.29	SI: cranial	G0C0**
		LR: right AP: ant	G270 C0**
5	0.93	SI: cranial LR: left AP: ant	G0C0** G90C0**
6	1.37	SI: medial LR: left AP: medial	G180C0 G210C0 G215C90
7	0.60	SI: medial LR: left AP: medial	G75C0** G130C0**
8	0.35	SI: medial RL: right AP: post	G270C0** G270C50**
9	0.99	SI: cranial RL: medial AP: ant	G90C0** G270C90**
10	3.22	SI: medial RL: right AP: ant	G50C0** G110C0**

**beam with range shifter.

All neuromas were deep seated and no pre‐absorber was needed for the treatment. The median delivered energy was 119 MeV (range 94–142 MeV), corresponding to a proton range in water of 10.5 cm (range 7.4–14.0 cm).

All meningiomas except one (patient #6) were superficial and a pre‐absorber was needed. The median delivered energy was 101 MeV (range 74–141 MeV). Taking into account that the water equivalent thickness of the pre absorber is 4.0 cm, this translates to a median proton range in the patient of 3.6 cm (range 0.5–9.6 cm) in water equivalent thickness.

Reducing the spot scaling factor from 1.0 to 0.5 increased the number of spots in the final plan by a factor of 5.8 ± 0.9. Correspondingly, the time length required for robust optimization increased about three times (from around 20 min to 1 h for the largest lesion treated).

For neuromas, a spot spacing scaling factor equal to 1.0 and 0.5 implied a distance between contiguous spots in the range of 5.1–5.9mm and 2.6–2.9 mm, respectively. For meningiomas, scaling factor equal to 1.0 and 0.5 implied a distance between a contiguous spot of in the range of 5.1–14.4 mm and 2.6–7.1 mm, respectively.

**FIGURE 1:**
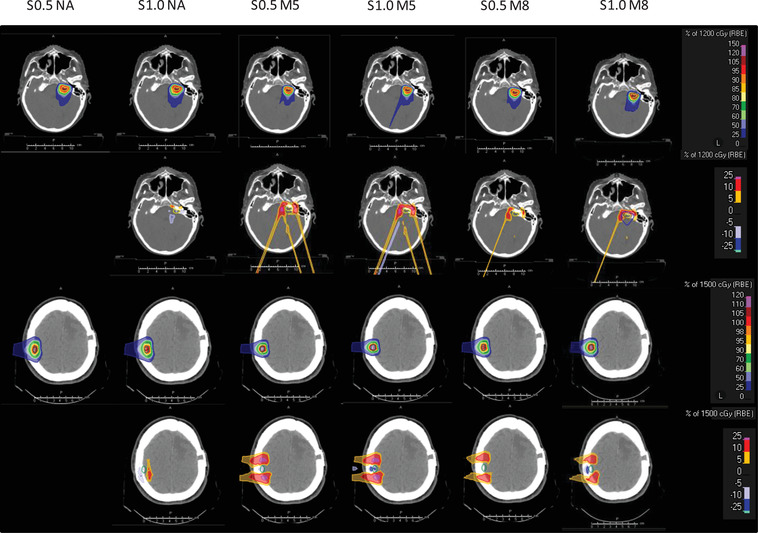
Dose distributions and dose differences obtained in a neuroma (top two rows) and a meningioma (bottom two rows) in the nominal scenario. The six plans are shown from left to right: S0.5 NA, S1.0 NA, S0.5 M5, S1.0 M5, S0.5 M8, and S1.0M8. The corresponding dose difference with respect to S0.5 NA is shown below

The dose distributions for a neuroma (patient # 1) and a meningioma (patient # 8) plan are shown in Figure 1, and the corresponding dose‐volume histograms for the target and the OARs are reported in Figures S1 and S2.

### 3.1 Target coverage

**FIGURE 2:**
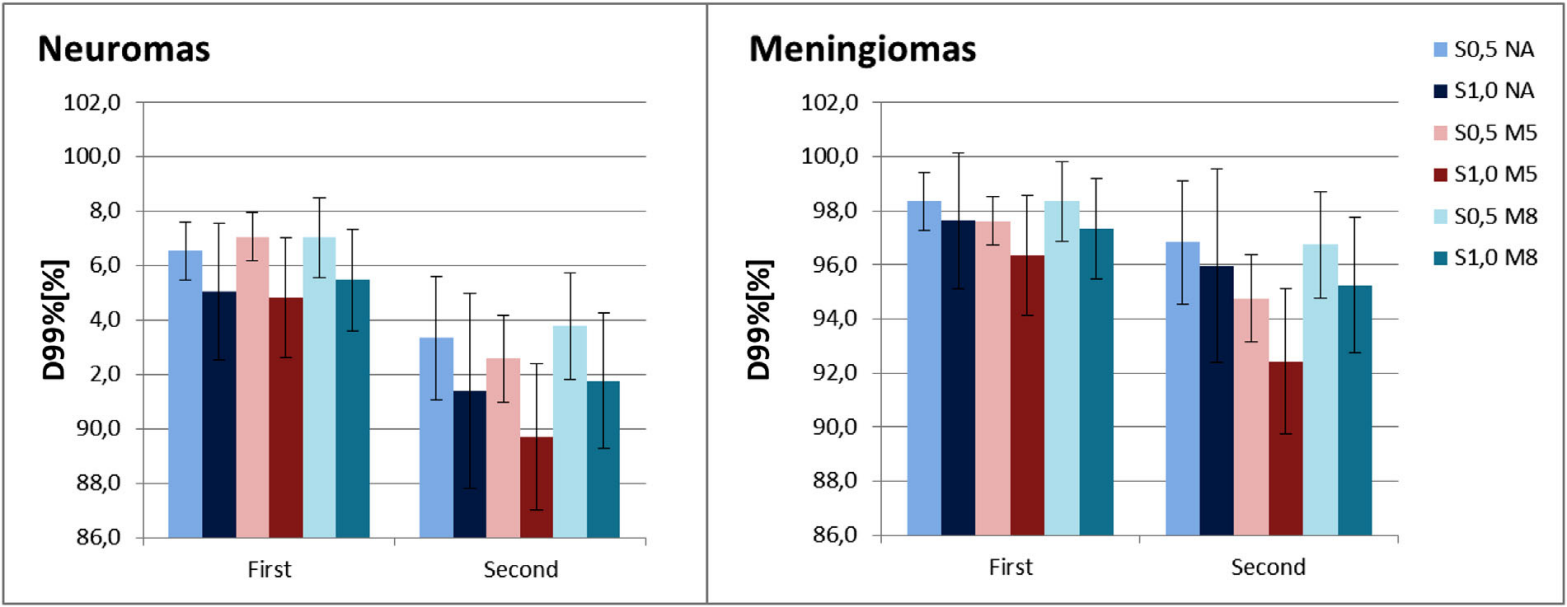
CTV D99% for neuromas and meningiomas in the two worst‐case scenarios. D99% is reported as percentage of D99% in the nominal plan, where 100% corresponded to the prescribed dose

The mean and standard deviations of worst‐case D99% (reported as percentage of the prescribed dose) of CTV, over all neuroma, and over all meningioma patients are presented in Figure 2. The values for individual patients are shown in Figure S3.

The impact of aperture on the robustness of CTV coverage was more pronounced in meningioma than in neuroma plans.

In neuroma plans, spot spacing and aperture did not have a statistically significant impact with the exception of the plans with 8 mm margin aperture, where the plan with smaller spot distance had a worst‐case scenario D99% 2% better than the plan with coarser spot spacing (*p* < 0.03).

In meningioma plans without aperture, the spot spacing did not significantly influence the target robustness. In the plans with apertures, a smaller spot spacing improved robustness (*p* < 0.01). The use of 5 mm margin apertures reduced robustness in both scenarios and for both spot spacing (*p* < 0.01). The use of 8 mm margin apertures did not significantly affect robustness in the first scenario for either spot spacings, or for the second scenario with a spot spacing factor of 0.5.

### 3.2 OAR sparing

**FIGURE 3:**
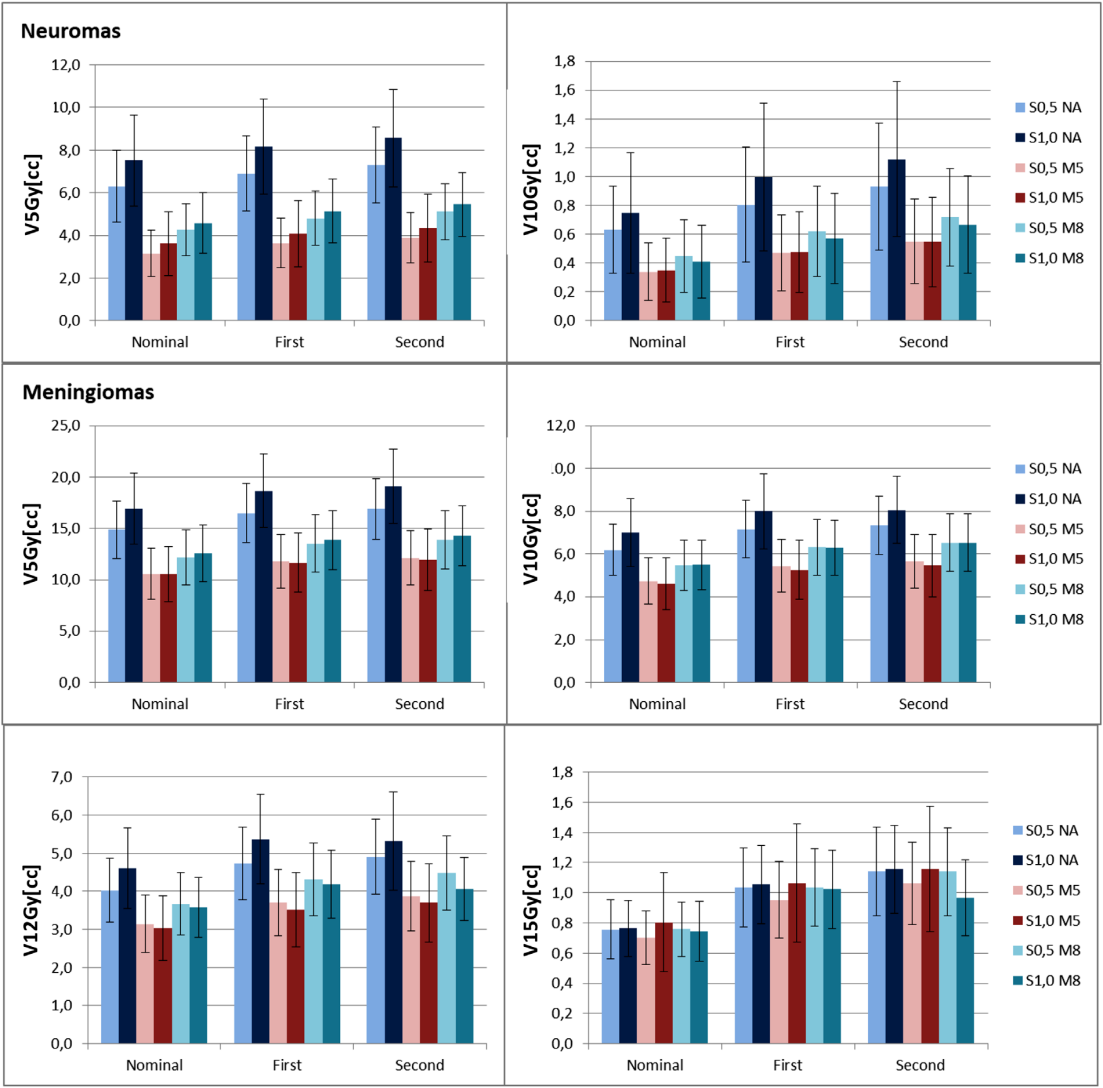
Healthy brain tissue dose in the nominal and perturbed scenarios. The colored bar shows the mean value for the patient population, and the error bars show the range

Figure 3 shows the mean and standard deviation of the dosimetric indices for the healthy brain tissue in the nominal, first, and second scenario. The corresponding values for the individual patients are shown in Figures S4 and S5. In neuroma plans V12Gy was never higher than 0.3 cc (patient #3, plan S1.0 NA, nominal scenario, 0.5 cc in the second scenario) and is not shown in the bar plots.

In neuroma plans, increasing spot spacing did not significantly affect the dose to the healthy brain tissue. Using apertures with 5 and 8 mm margin reduced V5Gy by around 50% (*p* < 0.01) and 32% (*p* < 0.02), respectively. A similar reduction was found in V10Gy but with a lower statistical evidence (*p* = 0.06–0.07). No significative reduction was found in V12Gy.

In meningiomas, tighter spot spacing reduced V5Gy only when apertures were not present (*p* < 0.03); V10Gy, V12Gy, and V15Gy were not affected. Apertures with 5 and 8 mm margin reduced V10Gy (by about 23% and 12%) and V12Gy (by about 22% and 9%), but did not significantly affect V15Gy.

### 3.3 Gradient index

**FIGURE 4:**
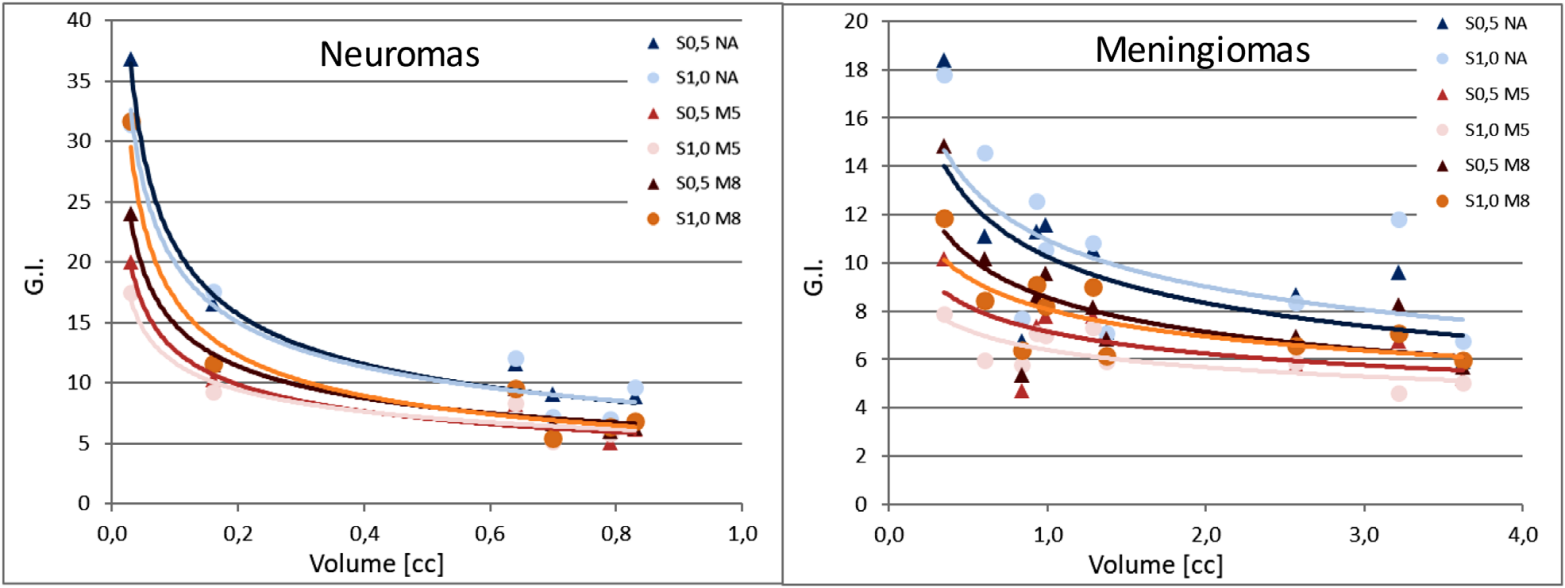
Gradient index (GI) is a function of the target size for neuromas (left) and meningiomas (right) for the six different plans. The continuous line is a fit of the data with a power function *y* = *ax* ^b^

The GI was related to the size of the lesion and is shown in Figure 4. The use of apertures improved the GI and reduced the low and medium doses to the healthy brain. The GI further improves with the use of aperture with smaller margin and with the increasing size of the lesion.

## 4 DISCUSSION

We investigated the influence of spot spacing, apertures, and their corresponding margin from the CTV on the plan quality in PBS treatment of neuromas and meningiomas. Robustness of target coverage and OAR sparing were analyzed.

While spot size depends on machine and beam line properties and it cannot be modified during treatment planning, spot spacing can be adjusted. Although in neuromas spot spacing did not affect OAR doses or the robustness of CTV coverage, in meningiomas a smaller spot spacing produced a reduction in V5Gy and an improved robustness in CTV coverage.

The use of apertures has been shown to be associated with a reduction in the OAR doses for different tumor sites. Su et al. investigated their impact in the treatment of the chest wall,^12^ concluding that the use of apertures improved the dose conformity to the target. Yasui et al. investigated the treatment of head and neck target at shallow depth,^13^ showing a small effect of apertures in the reduction of the maximum dose for OARs close to the CTV and a decrease of the volume receiving 50% of the prescribed dose by more of 10%. Rana et al.^14^ showed, in head and neck plans, that the use of beam‐specific apertures did not deteriorate the coverage and homogeneity and allowed for a reduction in mean dose to the OARs with an average difference ranging from 2.9% to 12.1%.

However, Moteabbed et al.^5^ previously highlighted that apertures, increasing conformity and dose falloffs, could make plans more susceptible to loss of robustness. Even though the use of apertures in neuroma plans reduced OAR dose but did not impact significantly on target robustness, in meningioma our findings support the hypothesis of those last authors. In fact, aperture of 5 mm margin reduced low and medium brain tissue doses in meningiomas but caused a reduction in plan robustness in the perturbed scenarios. An aperture with 8 mm margin produced an intermediate solution, with a smaller reduction in brain tissue doses but also a plan robustness which remained unaffected in the 0.5 mm perturbed scenario.

The dose fall‐off outside the target region was quantified by the GI, which improved when apertures were present, when a smaller margin was set and with larger lesions. The values we found were higher than the typical GI reported for photon treatment and the values reported for proton treatment by Cao et al.^15^ However, the size of the lesion investigated in that work (3.4–35.4 cc) were larger than those investigated in the present study (0.03–3.6 cc).

In conclusion, the optimal aperture margin is a trade‐off between the robustness of target coverage and OAR sparing, and the dosimetric benefit is a function of the size and depth of the lesion to be treated. In particular, the main impact of apertures was on low and medium doses to healthy brain tissue. In the treatment of neuromas and meningiomas, the use of aperture would reduce only doses lower than 12 Gy(RBE), a parameter which is poorly associated with radiation‐induced brain necrosis.^16^ In addition, the use of apertures is associated with an increased workload for the preparation and QA of the apertures and to longer treatment times, due to the need of re‐entering the room at every field in order to insert the correct aperture.

The impact of aperture could be more significant for higher prescription doses, as in the treatment of brain metastases and arteriovenous malformations, where doses higher than 20 Gy(RBE) in a single fraction are typically prescribed. In that cases, the use of apertures has the potential to reduce the volume of healthy brain tissue receiving a dose around 15 Gy(RBE), and therefore to be associated with superior treatment outcomes.

It should be emphasized that the dosimetric advantages of using patient‐specific aperture become more significant for high doses and in clinical routine has to be weighed against the increase in treatment complexity.

## CONFLICT OF INTEREST

The authors declare no conflict of interest.

## AUTHOR CONTRIBUTIONS

Roberto Righetto, Francesco Fellin, Marco Schwarz, and Paolo Farace conceived of the presented idea. Daniele Scartoni, Dante Amelio, and Maurizio Amichetti performed delineation of all patients and Roberto Righetto the planning. Roberto Righetto and Paolo Farace wrote the manuscript with support from Marco Schwarz.

## Supporting information

Fig. S1 Dose volume histograms for the neuroma patient # 1, for the nominal scenario.

Fig. S2 Dose volume histograms for the meningioma patient # 8, for the nominal scenario.

Fig. S3. Robustness evaluation. Worst case scenario for D99% of CTV for neuroma (left) and meningioma patients (right), shift 0.5 mm (top left and top right, respectively) and patient shift 1.0 mm (bottom left and bottom right, respectively).

Fig. S4. V5Gy and V10Gy for healthy brain tissue in neuroma patients for the six plans. Nominal (top), first (middle) and second scenario (bottom left).

Fig. S5. V5Gy, V10Gy and V12Gy for healthy brain tissue in meningioma patients for the six plans. Nominal (top), first (middle) and second scenario (bottom left).

## Data Availability

The data that support the findings of this study are available from the corresponding author upon reasonable request.
