# Supplementary material for: Is it beneficial to use apertures in proton radiosurgery with a scanning beam? A dosimetric comparison in neurinoma and meningioma patients
Source: J Appl Clin Med Phys. 2021 Nov 9;23(2):e13459. doi: 10.1002/acm2.13459 (PMC8833271; doi:10.1002/acm2.13459)
Supplement: Supplementary file 2 — Fig. S2 Dose volume histograms for the meningioma patient # 8, for the nominal scenario. [file ACM2-23-e13459-s004.pptx]

## Slide 1
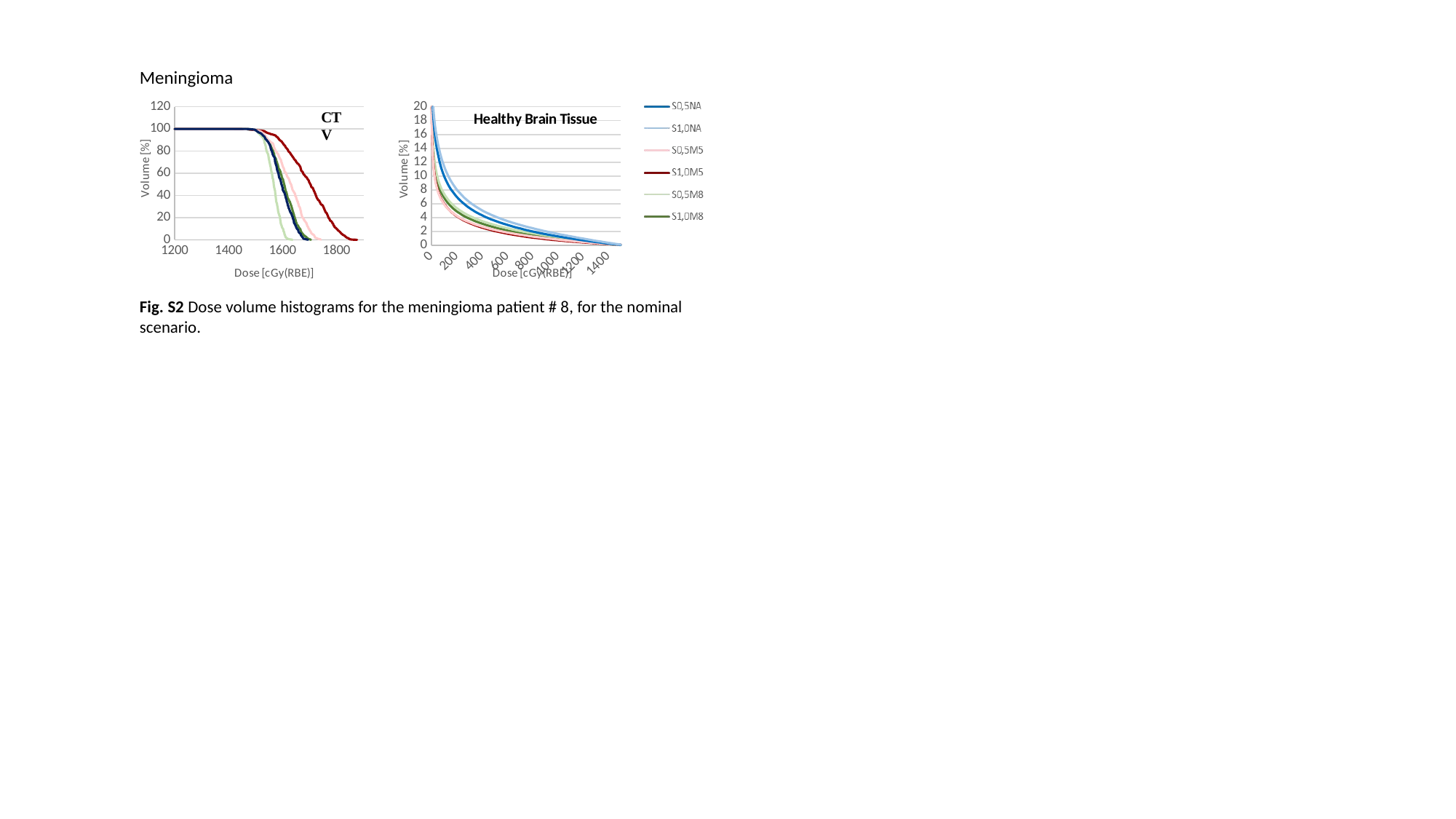

Meningioma
### Chart
| Category | S0,5NA | S1,0NA | S0,5M5 | S1,0M5 | S0,5M8 | S1,0M8 |
|---|---|---|---|---|---|---|
### Chart
| Category | S0,5NA | S1,0NA | S0,5M5 | S1,0M5 | S0,5M8 | S1,0M8 |
|---|---|---|---|---|---|---|Fig. S2 Dose volume histograms for the meningioma patient # 8, for the nominal scenario.
