# Supplementary material for: Is it beneficial to use apertures in proton radiosurgery with a scanning beam? A dosimetric comparison in neurinoma and meningioma patients
Source: J Appl Clin Med Phys. 2021 Nov 9;23(2):e13459. doi: 10.1002/acm2.13459 (PMC8833271; doi:10.1002/acm2.13459)
Supplement: Supplementary file 3 — Fig. S3. Robustness evaluation. Worst case scenario for D99% of CTV for neuroma (left) and meningioma patients (right), shift 0.5 mm (top left and top right, respectively) and patient shift 1.0 mm (bottom left and bottom right, respectively). [file ACM2-23-e13459-s001.pptx]

## Slide 1
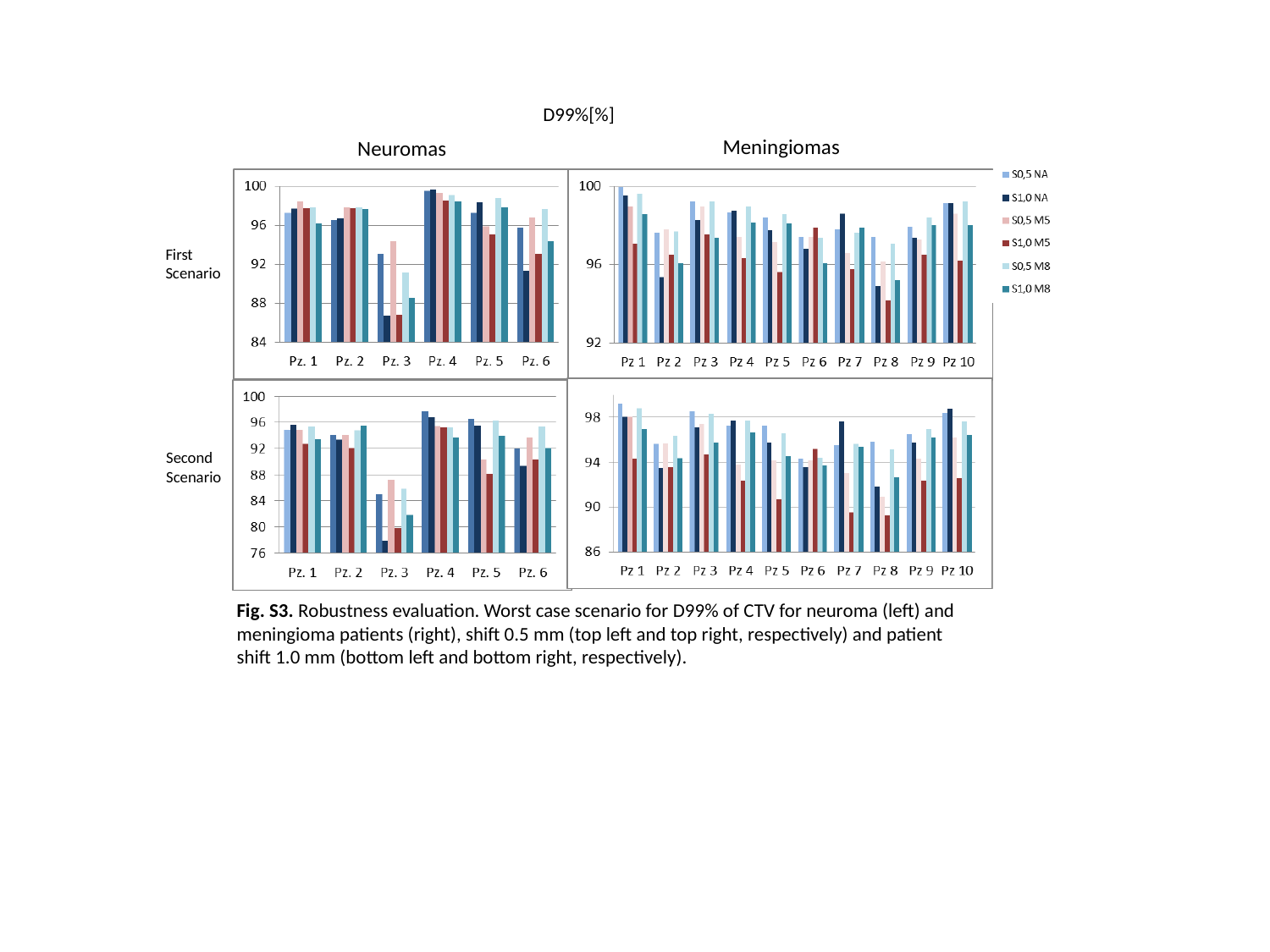

D99%[%]
Meningiomas
Neuromas
First Scenario
Second Scenario
Fig. S3. Robustness evaluation. Worst case scenario for D99% of CTV for neuroma (left) and meningioma patients (right), shift 0.5 mm (top left and top right, respectively) and patient shift 1.0 mm (bottom left and bottom right, respectively).
