# Supplementary material for: Is it beneficial to use apertures in proton radiosurgery with a scanning beam? A dosimetric comparison in neurinoma and meningioma patients
Source: J Appl Clin Med Phys. 2021 Nov 9;23(2):e13459. doi: 10.1002/acm2.13459 (PMC8833271; doi:10.1002/acm2.13459)
Supplement: Supplementary file 4 — Fig. S4. V5Gy and V10Gy for healthy brain tissue in neuroma patients for the six plans. Nominal (top), first (middle) and second scenario (bottom left). [file ACM2-23-e13459-s002.pptx]

## Slide 1
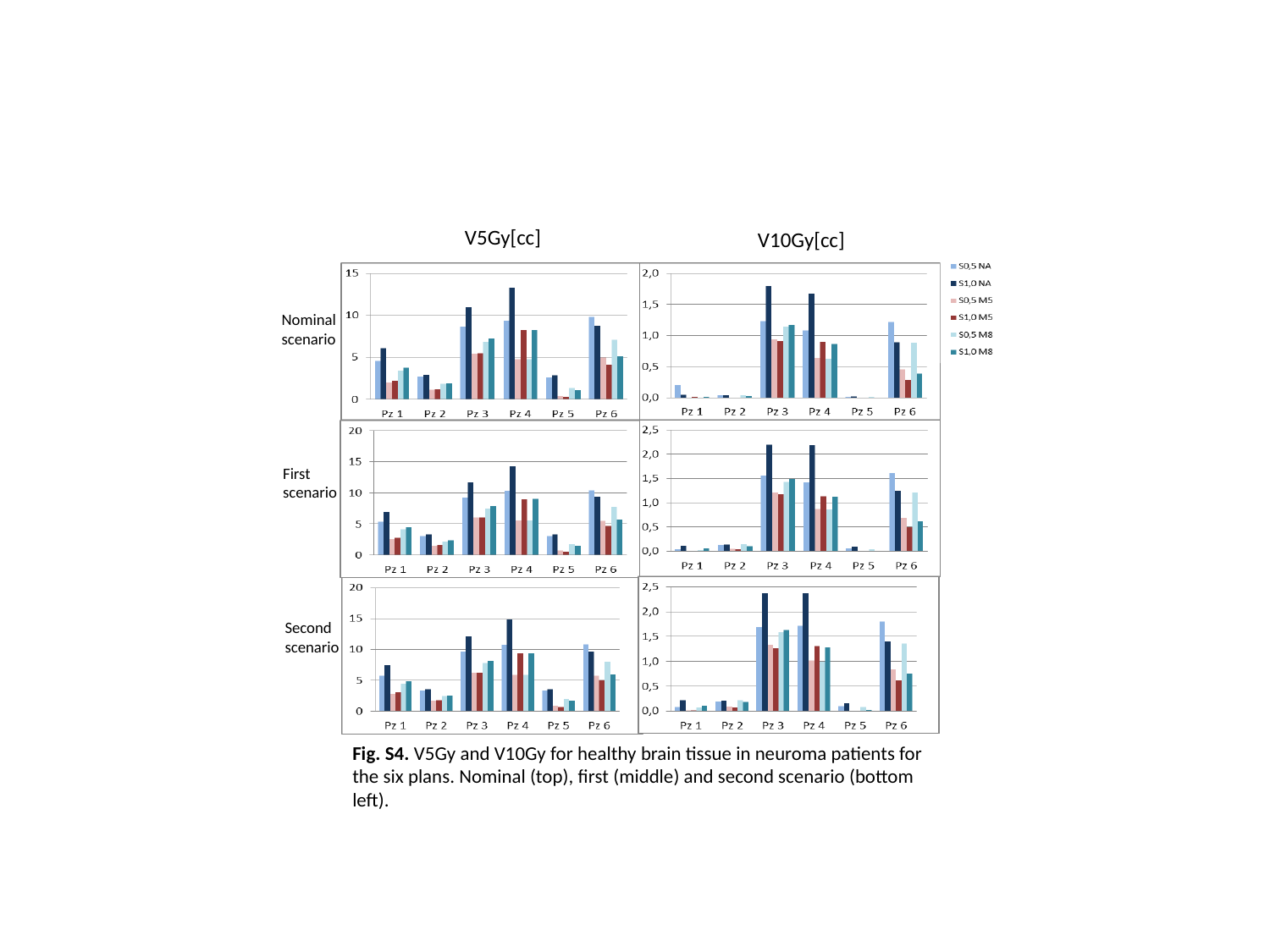

V5Gy[cc]
V10Gy[cc]
First scenario
Nominal scenario
Second scenario
Fig. S4. V5Gy and V10Gy for healthy brain tissue in neuroma patients for the six plans. Nominal (top), first (middle) and second scenario (bottom left).
