# Supplementary material for: Is it beneficial to use apertures in proton radiosurgery with a scanning beam? A dosimetric comparison in neurinoma and meningioma patients
Source: J Appl Clin Med Phys. 2021 Nov 9;23(2):e13459. doi: 10.1002/acm2.13459 (PMC8833271; doi:10.1002/acm2.13459)
Supplement: Supplementary file 5 — Fig. S5. V5Gy, V10Gy and V12Gy for healthy brain tissue in meningioma patients for the six plans. Nominal (top), first (middle) and second scenario (bottom left). [file ACM2-23-e13459-s005.pptx]

## Slide 1
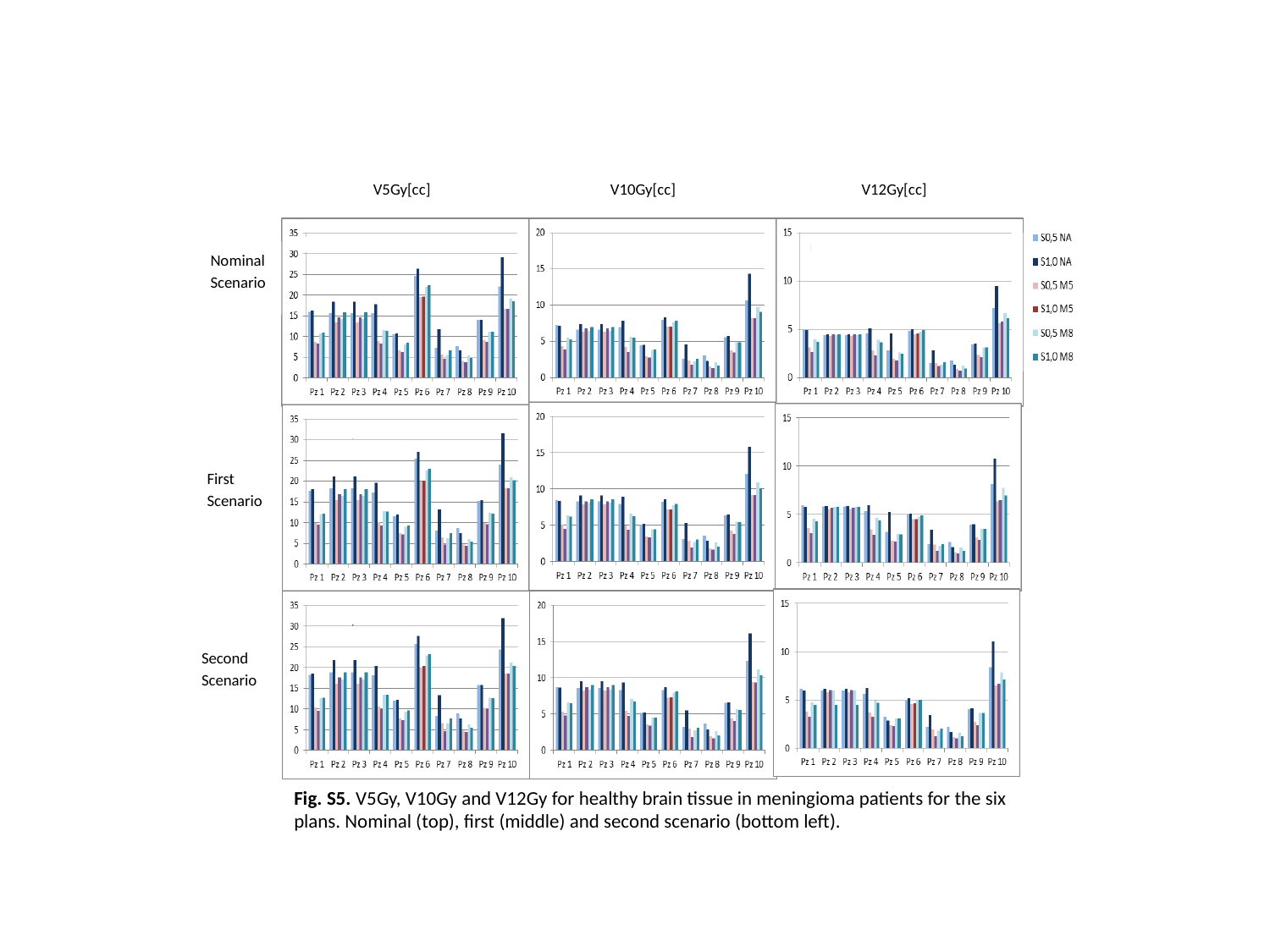

V5Gy[cc]
V10Gy[cc]
V12Gy[cc]
Nominal Scenario
First Scenario
Second Scenario
Fig. S5. V5Gy, V10Gy and V12Gy for healthy brain tissue in meningioma patients for the six plans. Nominal (top), first (middle) and second scenario (bottom left).
